# Compassion for midwives: The missing element in workplace culture for midwives globally

**DOI:** 10.1371/journal.pgph.0002034

**Published:** 2023-07-10

**Authors:** Kaveri Mayra, Christine Catling, Halima Musa, Billie Hunter, Kathleen Baird

**Affiliations:** 1 Birth Place Lab, Department of Family Practice, Faculty of Medicine, University of British Columbia, Vancouver, Canada; 2 Collective for Midwifery, Child and Family Health, School of Nursing and Midwifery, Faculty of Health, University of Technology Sydney, Sydney, Australia; 3 School of Healthcare Sciences, College of Biomedical and Life Sciences, Cardiff University, Cardiff, United Kingdom; The Aga Khan University, PAKISTAN and Judy Ngele Khanyola, University of Global Health Equity, RWANDA

By the very nature of caring work, it could be assumed that healthcare workers show compassion and respect for those they work with and care for. However, in many workplaces, respect, empathy, compassion and supportive teamwork are lacking. For compassionate workplaces to thrive, maternity care models, policies and environments need to be examined closely for factors that enhance or hinder this important aspect of healthcare. In this commentary, we discuss the importance of compassion ‘for’ midwives, as an essential, non-negotiable pre-requisite for compassion ‘by’ midwives.

Compassion, a highly valued attribute in midwifery [1], is defined as the ‘emotional response to another’s pain or suffering, involving an authentic desire to help’ [2]. Compassionate midwifery care may be endangered by ongoing staffing crises, relentless medicalisation of birth and high burnout levels [3]. When staff suffer from physical and emotional exhaustion and burnout, symptoms of detachment arise, compromising compassionate and effective care leading to compassion fatigue, moral injuries and long-term trauma [4]. Midwives enter the profession with great intentions, but often experience occupational dissatisfaction that leads them to leave [5].

A challenging workplace environment creates toxic team cultures, characterised by incivility and bullying behaviours. In such non-compassionate environments, students and newly qualified staff are the most affected [6]. As the UK Ockenden report [7] cautions, workplaces with an ‘us and them’ culture can create fear and reluctance to escalate clinical concerns leading to unsafe practices, with women, newborns and families being adversely affected. For midwives’ own health and wellbeing and compassionate practice, it is crucial that compassion be shown to the midwives themselves through the creation of supportive and comfortable working environments.

Occupation-related distress is a shared burden among midwives, regardless of where they work, exacerbated by the country’s resources, and the composition of the healthcare system and policies [6, 8]. In the global south, midwives’ experience of professional, social, and economic barriers linked to gender discrimination, inequality and inequity are more pronounced [9], while the issues exist in the global north as well.

## Non-compassion is universal, cross-cutting and contextual

Socialisation for a maternity care environment and culture lacking compassion often begins during pre-service education, when midwives and nurses start experiencing inadequate working conditions and structural inequalities and discriminations. For example, in India, four years of grueling pre-service ‘training’ often accustoms students to a non-compassionate team culture, gender-based inequality and profession-based stigma, and power-based hierarchical imbalance leading to discrimination. This is derived from the embedded societal construct and context of the health system. Indian health governance contains entrenched remnants of colonialism, evident in patriarchal policies and health systems that are insensitive to the (female) frontline healthcare providers [8, 10].

Non-compassion manifests itself in discriminatory behaviours and practices through examples such as discrimination in water, sanitation and hygiene resources in India and Nigeria, based on the co-authors’ experiences. This is shown through the lack of clean water and the only functional and clean toilet, often locked, and being located in the medical superintendent’s room, inaccessible to nurse-midwives. Therefore, staff are forced to urinate out in the open, behind bushes, or in isolated unsafe areas. Alternatively, they delay urinating until the end of 8–12 hour shifts. Historically, such a dehumanizing display of non-compassion through restricting access to water and sanitation has been used globally to create caste, class, religion, gender and race-based divisions and hierarchies [11].

Most global south countries face midwifery workforce shortages, inadequate maternity facilities and inadequate numbers of midwifery graduates [8]. To cope with excessive workload, in Nigeria midwives described improvising by wearing five gloves when attending to five births, removing the used gloves between women [12]. Similar to the global north, the resulting lack of ability to provide quality care due to burnout and moral distress influences midwives’ decision to leave the workforce [9, 12]. Increasing and unsustainable workload is an example of a structural challenge that demonstrates non-compassion and can lead to burnout.

Midwives experiencing workplace adversity and traumatic events risk developing secondary traumatic stress, post-traumatic stress disorder or depressive symptoms leading to compassion fatigue [13], affecting their ability to provide compassionate care [14]. Healthcare providers must consider how they enact compassion, and what language and tone they use, to ensure they do not silence women who need to reach out and ask for compassionate care and support.

There is often a dissonance between expressed empathy and societal norms in the global south. Empathy can be defined as the ability to identify with another person’s situation or feelings, whereas compassion is the response to empathy. While empathy is taught in the midwifery curriculum, practicing it in the clinical setting is often considered weak and undignified. The strictly disciplined school education in many countries in the global south aims to ensure that female students maintain composure and appear ‘dignified’ in their society [10]. The key issue is the dissonance between the ideals of working compassionately, in partnership with women and the realities of practice where the needs of the obstetric institution come first.

## Adding compassion to the work culture

Midwives are expected to provide sensitive care to women at times of joy, sadness, physical and emotional pain, including stillbirth, gender-based violence and sexual assault, and birth trauma. However, midwives are not empty vessels. They enter the profession of midwifery with personal histories that influence who they are, and ultimately how they behave as a midwife; their subconscious memories can affect their ability to be compassionate and empathetic.

The UK champion of compassionate leadership, Michael West, proposes three core needs critical for midwives’ and nurses’ wellbeing and motivation: autonomy, sense of belonging and ability to contribute [15]. The maternity care evidence supports West’s proposition: emotional wellbeing is enhanced when midwives work in emotionally supportive environments, where they feel valued and respected by colleagues and where they have an authentic sense of agency [12, 15]. Relational models, such as midwife-led care and continuity of care, facilitate compassion; when midwives know the women they care for, and vice versa, communication and trust can blossom [15].

To create compassionate work environments, we need to listen to midwives’ needs and experiences on what works and what doesn’t. In the global south, it could be ensuring clean and spacious changing rooms, and access to water and restrooms. Crucially, studies of midwives’ workplace wellbeing show that creating compassionate cultures should not be the sole responsibility of individuals. Whilst healthcare workers need to cultivate self-awareness and compassion for themselves and others, this should be within the context of a health system that firstly values compassion itself.

Systems change will not occur overnight, but intermediate steps can be taken. Examples such as Schwarz rounds, that allows structured staff discussions of emotional aspects of healthcare, can potentially enhance awareness of oneself and one’s colleagues, and clinical supervision can provide deep reflection on professional issues and support clinicians’ wellbeing. We also know the importance of providing support at times of greatest vulnerability, for example for newly qualified midwives [9] and for those who have experienced traumatic situations [14].

Compassionate care requires colleagues to be sensitive to each other’s wellbeing in a working environment with sensitive health policies and systems. A strong political will, through a wide consultative framework and investment in research, will enhance understanding of how to create compassionate workplaces. Most importantly, it requires leaders and policymakers who understand the value and importance of bringing ‘compassion’ back to maternity workplace culture.
